# Single-Entity Detection With TEM-Fabricated Nanopores

**DOI:** 10.3389/fchem.2021.664820

**Published:** 2021-05-07

**Authors:** Hongcheng Yang, Muhammad Saqib, Rui Hao

**Affiliations:** Department of Chemistry, Southern University of Science and Technology, Shenzhen, China

**Keywords:** single entity detection, sequencing, solid-state nanopores, TEM fabrication, electron-beam drilling

## Abstract

Nanopore-based single-entity detection shows immense potential in sensing and sequencing technologies. Solid-state nanopores permit unprecedented detail while preserving mechanical robustness, reusability, adjustable pore size, and stability in different physical and chemical environments. The transmission electron microscope (TEM) has evolved into a powerful tool for fabricating and characterizing nanometer-sized pores within a solid-state ultrathin membrane. By detecting differences in the ionic current signals due to single-entity translocation through the nanopore, solid-state nanopores can enable gene sequencing and single molecule/nanoparticle detection with high sensitivity, improved acquisition speed, and low cost. Here we briefly discuss the recent progress in the modification and characterization of TEM-fabricated nanopores. Moreover, we highlight some key applications of these nanopores in nucleic acids, protein, and nanoparticle detection. Additionally, we discuss the future of computer simulations in DNA and protein sequencing strategies. We also attempt to identify the challenges and discuss the future development of nanopore-detection technology aiming to promote the next-generation sequencing technology.

## Introduction

The capability to achieve single-entity detection has provided considerable progress in medicine and science (Raveendran et al., 2020a). For instance, studying and observing one molecule at a time could unveil heterogeneities, richer information of molecular interactions/behavior, and capability for revealing fundamental dynamic processes that occur at the interfaces of medicine, chemistry, biophysics, and life sciences (Miles et al., 2013; Raveendran et al., 2020b; Xue et al., 2020). Whereas, most of the traditional analytical methods provide population-based and time-averaged information, single-entity recordings enable us to reveal how an individual member of molecular populations interact and behave, resolving the spatial and structural dynamic processes. More recently, nanopores have emerged as the leading single-entity analytical tool for label-free, high-throughput, and low-cost characterization of individual protein molecules, nucleic acids, and nanoparticles (NPs), with a nanometer-sized hole or channel embedded in an ultrathin membrane that separates two chambers containing electrolyte solutions. Nanopore-based detection technologies originate from ion channels and Coulter counter invention (Shi et al., 2017).

Over the years, the field of nanopores is fueled by the ambition for commercialization of nanopore-based sensing and nucleic-acid sequencing/genome mapping applications (Deamer et al., 2016). Subsequently, over a dozen companies including Oxford Nanopore Technologies, Nanopore Solutions, Quantapore, Two Pore Guys, Genia Technologies, Hitachi, and so on, are pursuing the commercialization of the nanopore-based sequencing and sensing technologies (Jain et al., 2018; Amarasinghe et al., 2020). Nowadays, nanopore-sensing platforms are rapidly progressing in grand challenges of DNA sequencing, protein sequencing, diagnostics, and biological screening (Restrepo-Perez et al., 2018; Cai et al., 2019).

The practical applications of nanopores include detection of length, structure, and conformation of biomolecules, sequencing, single-molecule dynamics, counting as well as sizing of NPs. When a single molecule passes through the nanopore under an applied electric field, the variation in ion current signals could reveal dynamic motion and the structure of the molecule (Venkatesan and Bashir, 2011). The variation in ion current signals reveals the concentration and size of the molecule inside the nanopore when molecules pass through the ion channels (Chen and Liu, 2019). It also provides information about the dynamic translocation behavior of molecules.

Typically, nanopores can be broadly categorized into biological and solid-state nanopores based on materials. Biological nanopores are usually composed of protein structures (e.g., aerolysin and α-hemolysin) while organic or inorganic materials are utilized to fabricate the solid-state nanopores (e.g., glass tubes, polymeric films, two-dimensional nanosheets, anodic aluminum oxide (AAO) nanochannels; Zhu et al., 2018, 2020; Tang et al., 2019; Zhou et al., 2019). However, biological nanopores have inherent shortcomings of sensitivity to surrounding experimental conditions (e.g., temperature, pH), poor mechanical stability, and fixed pore size and shape (Chen and Liu, 2019). In contrast, solid-state nanopores have many advantages, such as reusability, adjustable pore size, mechanical robustness, and stability in physical and chemical environments (Venkatesan et al., 2009, 2010, 2012; Schneider et al., 2010). Among these solid-state nanopores, introducing nanopores in the thin membranes, such as graphene (Arjmandi-Tash et al., 2016), silicon nitride (SiN_x_) (Dimitrov et al., 2010), silicon dioxide (SiO_2_) (Luan et al., 2012), aluminum oxide (Al_2_O_3_), and molybdenum disulfide (MoS_2_) (Graf et al., 2019) has been proved to be advantageous because of their easy modification, controllable pore size, adjustable membrane thickness, and surface charge. These unique characteristics could facilitate the enhancing of the spatiotemporal resolution of nanopore sensing and prevent biomolecular clogging in the pore.

Diverse techniques have been developed for nanopore fabrication on thin films, including chemical etching, dielectric breakdown methods, e-beam sculpting, focus ion beam, and electron beam drilling (Han et al., 2019). Among these methods, transmission electron microscopy (TEM) nanopore drilling is the most popular for thin membranes. It offers high resolution and a direct imaging mode to monitor the nanopore generation in real-time (Dekker, 2007). In particular, the high-energy electrons of the TEM significantly benefit the sputtering of a membrane to generate nanopore without residual impurity (Kim et al., 2011). Compared with ion beam methods, the e-beam methods have certain advantages, including finer control and lesser damage to the substrate (Yang et al., 2017; Horak et al., 2018; De Teresa et al., 2019). Moreover, a combination of high-resolution spherical aberration-corrected TEM (Cs-corrected TEM) leads to an improved (sub-nanometer scale) fabrication and characterization of the structures, geometries, and functionalization of nanopores at the atomic levels.

Despite the remarkable progress, many challenges associated with easy clogging in the pores, high translocation speed, and low signal-to-noise ratios (SNRs) are not completely resolved and shall necessitate further research (Wang et al., 2021). Therefore, in this mini review, we focus on the latest progress in fabrication, characterization, modification, and SNR enhancing strategies of TEM-fabricated nanopores. Current efforts to detect nucleic acids, protein molecules, and NPs with an emphasis on emerging fields are shown. Future research directions to overcome the challenges in nanopore-based single-entity detection are discussed.

## Fabrication, Characterization, and Modification of Nanopores

**Figure 1:**
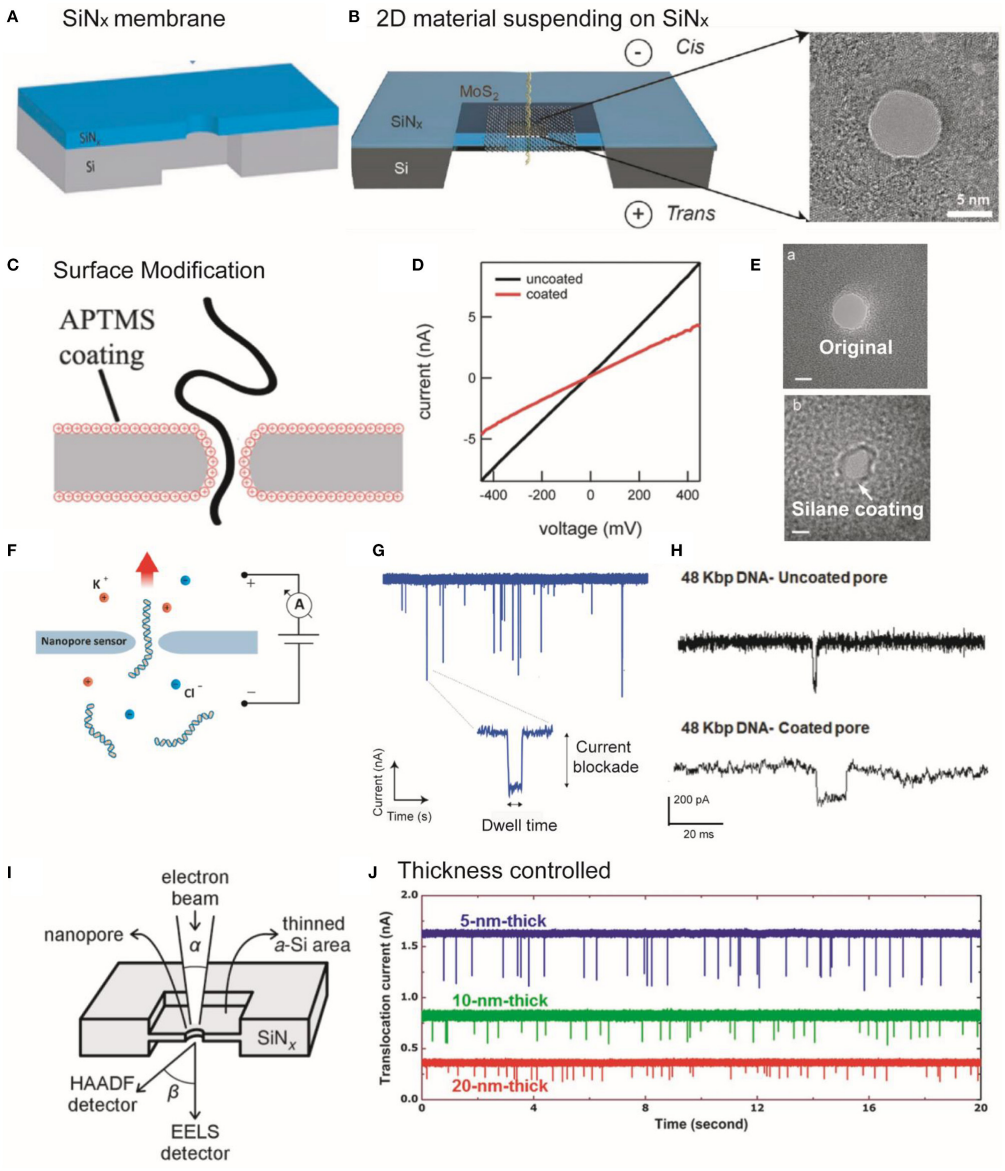
**(A,B)** Schematic illustration of SiN_x_ and MoS_2_ membrane suspended on a SiNx supporting membrane. **(A)** Reprinted from Wang et al. (2020) with permission from the Royal Society of Chemistry. **(B)** Reprinted from Liu et al. (2014) with permission from the American Chemical Society. **(C)** Schematic showing DNA threading through an APTMS-coated SiN_x_ nanopore. **(D)** *I–V* curves of coated and uncoated pores. Reprinted from Anderson et al. (2013) with permission from the American Chemical Society. **(E)** TEM images showing the original and silane-coated nanopores. Reprinted from Wanunu and Meller (2007) with permission from the American Chemical Society. **(F)** Image showing a nanopore that separates two chambers containing electrolyte solutions and small molecules are passed through the nanopore driven by the applied potential. **(G)** An amplifier showing the detection of the partial current blockade due to the passage of molecules through the nanopore. The translocation event of an individual molecule is usually characterized through dwell time and the amplitude of the current blockade. Reprinted from Fragasso et al. (2020) with permission from the American Chemical Society. **(H)** Example showing the DNA (48 kbp) translocation events through uncoated and DOPA-coated nanopore. Reprinted from Karmi et al. (2020) with permission from the American Chemical Society. **(I)** Diagram of STEM-thinning method. The electron beams are collected using electron energy-loss spectroscopy (EELS) and HAADF signal detectors after interaction with the film. Reprinted from Rodriguez-Manzo et al. (2015) with permission from the American Chemical Society. **(J)** The translocation signals were enhanced in thinner membranes with the translocation of 40 nt ssDNA through nanopore, providing the higher SNR for nanopore sensing. Reprinted from Lee et al. (2014) with permission from Springer Nature. APTMS, 3-(aminopropyl)trimethoxysilane; TEM, transmission electron microscope; DOPA, amino l-3,4-dihydroxyphenylalanine; STEM, scanning transmission electron microscopy; EELS, ssDNA, single-stranded DNA; SNR, signal-to-noise ratio; HAADF, high-angle annular dark-field.

TEM, with the high-resolution imaging properties, affords a stable performance to fabricate and characterize nanopore structures. The adjustable electron beam sizes, electron doses, and accelerated voltages of TEM drilling significantly improved its application to achieve controllable nanopore sizes (Storm et al., 2003; Xu et al., 2012; Yuan et al., 2020). The scanning transmission electron microscopy (STEM) mode is crucial for controlling the electron irradiation doses and reducing the electron beam sizes (Xu et al., 2013). Cs-TEM has been used to drill the nanopore with a size <2 nm due to the smaller beam size as compared with that without the spherical aberration correction properties. Moreover, TEM is the major tool to drill nanopore in the atomic thickness two-dimensional (2D) materials (graphene, boron nitride (BN), MoS_2_, and MXenes) suspended on the SiN_x_ membranes (Figures 1A,B; Garaj et al., 2010; Petrone et al., 2012; Liu et al., 2014; Nehra et al., 2019).

The specific nanopore shapes on SiN_x_ membranes, such as cylindrical (Chen et al., 2004), conical, and hourglass (Venkatesan et al., 2009, 2010; Maitra et al., 2012) are important to functionalize receptors inside the nanopore (Tabard-Cossa et al., 2007; Maglia et al., 2010). Based on the energy distribution of electron beam, the TEM-fabricated nanopores delivered laterally symmetric structures, namely an hourglass shape (Kim et al., 2006, 2007) or a cylindrical structure. Hout et al. revealed that narrow beam size produces cylinder-like nanopores, while the wider beam generates compressed hourglass-like nanopores (van den Hout et al., 2010; Hu R. et al., 2020). Furthermore, TEM tomography can provide three-dimensional (3D) analysis of inner structure. The electron energy-loss spectroscopy (EELS) can analyze low atomic number elements (C, N, O, B), valence states of elements, and deficient regions with nanometer-scale resolution (Kim et al., 2006; Si et al., 2018; Chou et al., 2020).

For single-entity detection, nanopores must be a stable, robust, reliable, and smooth surface. The optimal design should also prevent the expansion, shrinkage, and the non-specific absorption of nanopores. The high salt solutions or polar solvents could induce the expansion or shrinkage of unmodified SiN_x_ nanopores (Li et al., 2012), which can be solved with the inorganic materials coating (Feng et al., 2015). The hydrophobicity of the nanopores could induce biomolecules sticking and clogging in the rough nanopore wall (Schneider et al., 2013; Tang et al., 2014; Liang et al., 2015) leading to disturbed signals and damage to the device (Niedzwiecki et al., 2010). With antifouling modification, such as lipid bilayer and polyethylene glycol coating, the drawback can be dealt with (Balan et al., 2014; Tang et al., 2014; Shekar et al., 2016).

The surface chemistries (modification) of nanopores play critical roles in electro-osmotic flow, receptor binding, pore–analyte surface interaction, and translocation process (Wei et al., 2012). To tailor the surface properties, several types of strategies have been reported. For instance, SiN_x_ or glass made nanopores can be modified using silane chemistry, whereas metallic (e.g., Au) nanopores can be modified by applying thiol chemistries via solution or vapor-based depositions (Yin et al., 2017). Likewise, several monolayer agents have been used to functionalize the surface (e.g., chemical groups, roughness, and surface charge) of solid-state nanopores including cetyltrimethylammonium bromide, Tween 20, polyethylene glycol (PEG), organosilanes via non-covalent and covalent bonding interactions (Giamblanco et al., 2018; Eggenberger et al., 2019; Awasthi et al., 2020). For example, Meller group used 3-(aminopropyl)trimethoxysilane (APTMS) to modify the surface of SiN_x_ nanopores (Figures 1C,D; Anderson et al., 2013). They showed that conductance measurements can be used to determine the size of the pores (Figure 1D; Anderson et al., 2013), but does not specifically reflect the geometries of the nanopores (Kowalczyk et al., 2011; Wang et al., 2018). Surface modification can decrease the nanopore size (Figure 1E; Wanunu and Meller, 2007).

Lipid bilayers can be applied to coat graphene, quartz, and Al_2_O_3_ materials (Yusko et al., 2011; Eggenberger et al., 2019). Another tactic is to attach protein channels to the nanopores, merging the superiorities of both biological and solid-state nanopores. The hybrid nanopore formation have been demonstrated using α-hemolysin (α-HL) (Hall et al., 2010) viral portal proteins and DNA origami (Bell et al., 2012). The surface modification strategies can enhance nanopore selectivity via two ways: (1) the functionalization of the nanopores with a receptor designed for a specific target (e.g., aptamers, single-protein receptors) and (2) the introduction of probes (in solutions) those can selectively capture target molecules (Iqbal et al., 2007; Bell and Keyser, 2016; Sze et al., 2017).

Recently, solid-state plasmonic-nanopore devices are emerging as superior single-molecule sensing tools due to their several advantages (Spitzberg et al., 2019). The mass production of the solid-state nanopores and pore formation in a uniform (small) size/structure is still a challenging task with the TEM-based methods. Therefore, stable and high-throughput fabrication techniques should be optimized and developed to improve the reliability and sensitivity of the nanopore-based single-entity detections.

## The Detection Mechanism and the Enhancement of SNR Strategies for Nanopore Sensing

To design the detection setup, a thin membrane with a nanopore separates the electrolyte solutions into two compartments, and a voltage is applied across the membrane (Figure 1F). When the target analyte passes through the nanopore, each type of the single entities can transiently block the ionic current, thereby generating a characteristic current drop signal with current blockage (Δ*I*), dwell time (*t*_d_), and frequency (capture rate, *R*_c_), which is detected by an analog-digital converter and patch-clamp amplifier (Figure 1G; Dekker, 2007; Fragasso et al., 2020). As a result, the sizes, lengths, shapes, and conformations of the single entities can be discriminated by analyzing the peak shapes, dwell times, capture rates, and full width half maximum (FWHM) values of the relative current blockade distribution. Moreover, amino l-3,4-dihydroxyphenylalanine (DOPA)-based modification can significantly improve the SNR and durability (up to months) and reduce (an order of magnitude) the DNA translocation as compared with that of uncoated nanopore (Figure 1H; Karmi et al., 2020).

The development of noise reduction techniques is essential to sensitively discriminate against single-molecule structures and enhance detection resolution (Maitra et al., 2012). For example, the thickness of the membrane limits vertical sensing resolution. To resolve this problem, Rodriguez-Manzo et al. employed a STEM-thinning method (Figure 1I) to fabricate SiN_x_ nanopores (<2 nm thick) (Rodriguez-Manzo et al., 2015). As shown in Figure 1J, the thinner SiN membranes can achieve high vertical resolution and SNR (Lee et al., 2014). Since DNA base pairs exhibit a gap of ~0.3 nm, a ~0.3-nm-thick membrane is required to avoid the reading of multiple nucleotides at the same time. A graphene sheet and other 2D materials with atomic thickness, such as MoS_2_, BN (Liu et al., 2013), and MXene (Mojtabavi et al., 2019) can be used for sequencing with high vertical spatial resolution. Moreover, significant efforts have been devoted to reducing electrical noise, including amplifier noise, dielectric noise, thermal noise, and 1/f noise (flicker noise) (Lee et al., 2018).

The molecule translocation velocity through a nanopore is influenced by several key parameters, including temperature, surface charge, ion concentration, solvent viscosity, and pore size (Anderson et al., 2013; Larkin et al., 2013; Liang et al., 2014; Banerjee et al., 2015; Sha et al., 2017). Retarding biomolecule translocation could also increase sensitivity (Fologea et al., 2005). The translocation speed through the pore is in the range of 3,000–50,000 nucleotide (nt) ms^−1^. Consequently, this fast speed hinders their capability to achieve single-nucleotide resolution in DNA sequencing. To this end, Feng et al. exploited a room-temperature (high viscosity) ionic liquid (RTILs) to regulate the DNA translocation speeds (1–50 nt ms^−1^) through MoS_2_ nanopores. They showed that single nucleotides can be identified in ultrasmall (narrow orifices) MoS_2_ pores by controlling the dynamics of DNA translocation (with RTIL), while retaining an SNR >10 (Feng et al., 2015). The SNR can also be enhanced by improving the molecular specificity, eliminating clogging and non-specific adsorption, and enhancing the temporal and vertical resolutions in solid-state nanopore sensing (Chou et al., 2020).

## Applications

### DNA Detection and Sequencing

The full-length reads of DNA strands and other advantages brought by the latest innovations in nanopore sensing offer unprecedented opportunities to analyze DNA sequence and its fragment sizing based on the characteristic current drop signals. Besides, nanopores can be used to distinguish single- and double-stranded DNA (dsDNA) (Schneider et al., 2013) and complex DNA knots (Kumar Sharma et al., 2019). By controlling the degrees of nanostructuring, biodetection sensitivity, and dynamic range of sensing systems can be significantly improved (Soleymani et al., 2009). Due to its powerful characteristics, nanopore DNA detection and sequencing has a strong potential in improving genetic diagnoses and elucidating numerous disease mechanisms.

**Figure 2:**
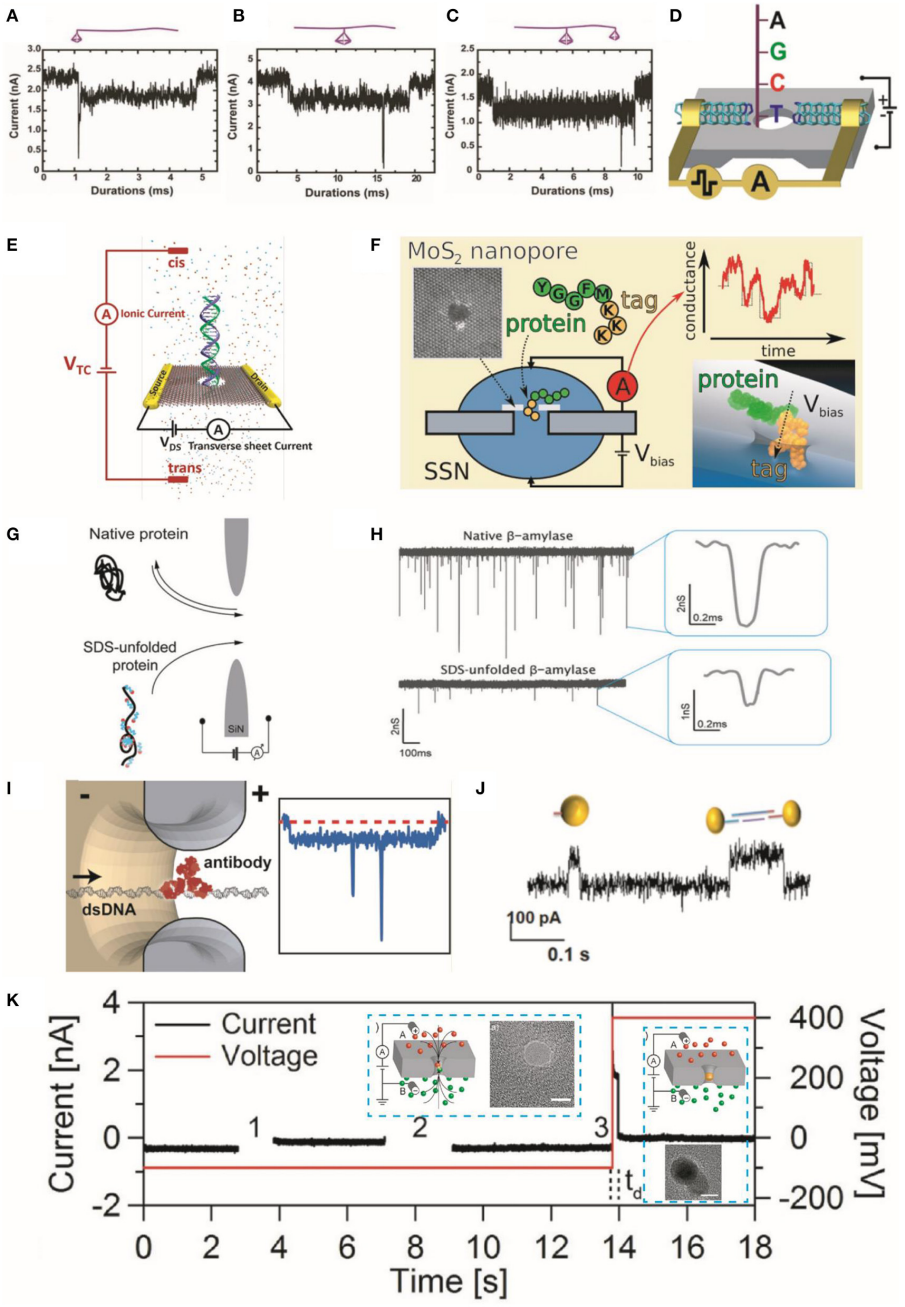
Characteristic translocation current blockade signals for representative structures of TDN bonded to linear DNA molecules. **(A)** One end bonded to a 7-bp TDN, **(B)** the middle part bonded to a 13-bp TDN, and **(C)** a 13-bp and a 7-bp TDN bonded to the middle and end, respectively. Insets are cartoon images of these structures. Reprinted from Zhao et al. (2019) with permission from the Royal Society of Chemistry. **(D)** DNA sequencing setup showing transversal current rectification during translocation of ssDNA through a nanopore with N-terminated CNT electrodes. Reprinted from Djurišić et al. (2020) with permission from the American Chemical Society. **(E)** Schematic showing the detection of dsDNA in electrolyte using transverse current and ionic current measurements. Reprinted from Xiong et al. (2020) with permission from the American Chemical Society. **(F)** Schematic showing the translocation of a model protein (peptides) and lysine residues through MoS_2_ nanopores with polylysine tags. The right panel shows the relationship between the translocation sequence of events and ionic conductance. Reprinted from Nicolai et al. (2019) with permission from the American Chemical Society. **(G)** Illustration of translocation events of native β-amylase protein and their sodium dodecyl sulfate-unfolded treatment structures through nanopore. **(H)** Current signals generated by the translocation of protein molecules; wherein the right panel displays their characteristic events. Reprinted from Restrepo-Perez et al. (2017) with permission from the Royal Society of Chemistry. **(I)** Illustration representing the translocation of a dsDNA molecule with a bound antibody (left panel) and corresponding spike responses. Reprinted from Plesa et al. (2015a) with permission from the American Chemical Society. **(J)** Current traces of AuNP translocation through the pore. Reprinted from Karmi et al. (2021) with permission from the American Chemical Society. **(K)** Schematic of the AuNP growth in nanopore; (left panel inset) the voltage difference triggers the reaction and drives the reagents into nanopore (right panel inset) the formation of a AuNP further blocks the pore and prevents the mixing of reagents. Current or voltage vs. time traces representing a particle growth procedure into three steps. Zero current indicates the NP formation. The time delay (*t*_d_) before formation of AuNP is represented with vertical [after step (3)] dashed. Inserts are the TEM images of nanopore before/after the AuNP growth. The scale bar is 5 nm. Reprinted from Venta et al. (2013) with permission from the American Chemical Society. TDN, tetrahedral DNA nanostructure; ssDNA, single-stranded DNA; CNT, carbon nanotube; dsDNA, double-stranded DNA; AuNP, gold nanoparticle; NP, nanoparticle; TEM, transmission electron microscope.

This emerging field of research usually involves engineering of dsDNA with additional protein-binding sites (Albrecht, 2019). As shown in Figures 2A–C, Zhao et al. demonstrated that tetrahedral DNA nanostructures could produce additional durations with sharp spikes in the ionic current signals when they bound to linear DNA molecules (Zhao et al., 2019). Similarly, Plesa et al. showed that a protrusion in the main DNA strand could produce a characteristic secondary current drop along with DNA strand translocation signals (Plesa et al., 2015b). Therefore, nanopore sensing can detect the complex DNA structures based on characterized spike peaks along with typical blockage current signals of DNA. Bashir and Wanunu groups have reported methods to detect DNA methylations (Shim et al., 2013) and discriminate pyrimidines (Henley et al., 2015).

As the next-generation sequencing technologies, the goal of the nanopore sensing is to discriminate between A–T and G–C base pairs in DNA strands. According to the previous investigations, the single nucleotides of A, T, G, and C can be identified based on their distinct transient residence time in the atomically thin MoS_2_ nanopore using a viscosity gradient system for reducing their translocation speeds (Chou et al., 2020). However, major challenges still exist in sequencing DNA strands with solid-state nanopores. To tackle these challenges, computer simulation methods can assist us in investigating DNA–nanopore interactions and reveal atomic-level detail of DNA translocation dynamics. Using molecular dynamic (MD) simulations, Farshad and Rasaiah showed that the signals of transverse and longitudinal ionic currents can be applied to identify four types of nucleotides (Farshad and Rasaiah, 2020). The introduction of neural network machine learning has achieved an over 80% accuracy to classify the A, G, T, and C in homo-oligonucleotides. Therefore, adapting software/open-source tools and MD simulation/computational resources holds strong potential in promoting nanopore DNA sequencing (Editorial, 2018).

Djurišić et al. achieved strong transversal current rectification of single-stranded DNA in N-terminated carbon nanotube (CNT) electrodes (Figure 2D) based on non-equilibrium (finite bias) Green's function (NEGF) + density functional theory (DFT). They developed a new sequencing approach with high nucleobase specificity (Djurišić et al., 2020). Likewise, the Leburton group used a combined theoretical-experimental method to analyze the variations (resistive effects) of the transverse current response during the translocation of protein and DNA molecules (Figure 2E) through a MoS_2_ membrane nanoribbon (Xiong et al., 2020). Further improvements can be made through greater computer resources, machine learning algorithms, and larger databases to improve the classification accuracy of base pairs.

Over the years, intensive efforts have been made to improve the performance (e.g., throughput, accuracy) of the solid-state nanopores in DNA detection and sequencing. Using advanced modification technology and genomic design, sensitive detection of miRNAs, small/multiple proteins, and antigens has been achieved using nanopores and DNA complexes. Nevertheless, some key issues such as control of the translocation velocity (translocation dwell time), non-specific interaction between the nanopore wall and biomolecules, and recognition at a single-nucleotide level still need to be addressed. With the further enhancement of the reliability and sensitivity of solid-state nanopore (genomic) technologies, new opportunities in clinical diagnosis (practical sequencer) and accurate detection of modified DNA structures and DNA–protein complexes are expected.

### Protein Detection and Sequencing

Proteins play many crucial roles in regulating physiological functions and maintaining metabolism. With unique 3D structures, low charge density, and diverse charge distribution of amino acids, protein detection is more challenging as compared with that of DNA (Hu Z. L. et al., 2021). Most of the traditional techniques require a large number of identical protein samples, wherein it is challenging to record a single-protein conformational change in a real-time manner (Ding et al., 2019). The nanopore-based approaches can achieve sensitive and label-free detection of a single protein even at extremely low concentrations as well as provide a dynamic view of protein conformation, confirming their ability for protein characterization (Acharya et al., 2020).

Nanopore sensing can differentiate different (weight) protein molecules by the characteristic ionic current signals (Plesa et al., 2013; Yusko et al., 2017; Giamblanco et al., 2018). Yu et al. labeled the three similar peptide sequences with the cysteine residues modified with negatively charged moieties to identify their conformations by comparing FWHM values, relative current blockade, and capture rates (Yu et al., 2019). Nicolai et al. investigated the translocation of a model protein (peptides) and lysine residues through MoS_2_ nanopores with polylysine tags applying MD simulations (Figure 2F). They further analyzed and discussed the translocation sequence of events and ionic conductance (Nicolai et al., 2019).

Proteins naturally exist in folded form and irregular charge state, which restricts their applications in sequencing using nanopore technologies. Besides, the protein functions are decided by the conformation of amino acid and are important for proteomic studies (Si et al., 2019). Therefore, it is important to treat unfolded proteins and functionalize them with uniform charges before application in sequencing. To this end, Restrepo-Perez et al. utilized sodium dodecyl sulfate to enhance the negative charge of protein molecules. They revealed that this strategy exhibits a much lower current blockade signal as compared with that of native proteins because of their unfolded structure with linear form (Figures 2G,H; Restrepo-Perez et al., 2017). Dekker Group showed that individual DNA-bound proteins could be detected using solid-state nanopores. They introduced a new anti-DNA antibody (Figure 2I) model system to explore the transient interactions between the antibodies and pore and to visualize specific complexes (Plesa et al., 2015a). They also introduced atom force microscope (AFM) probe tips, which can discriminate the C and N terminals of amino- or carboxyl-groups in proteins across the nanopore. For instance, Si et al. utilized AFM to manipulate protein structures in terms of both permeation velocity and transport direction through the nanopore. They further validated their results with the assistance of MD simulations (Si et al., 2019). They demonstrated that their strategy could successfully discriminate against different protonation states of the amino acids.

Solid-state nanopores have made considerable progress for the real-time detection of protein conformations (single-protein molecules) and characterization, setting up the basis for drug screening and disease-related research. Solid-state nanopore technology can improve the sensitivity of existing single-molecule protein sequencing technologies without labeling. Despite the latest advancements, it is necessary to further enhance the temporal and spatial resolutions (both vertical and lateral), control the protein translocation speed, and achieve low noise levels. With the developments of unfolded treatment strategies, novel device designs, preparation of new materials, controllable movement through nanopore, selectively capturing tools, and with the assistance of computer simulations, nanopore technology will continue to capture the complicated conformational and sequencing behavior of proteins.

### NPs Detection and Other Applications

Fragment sizing, sequencing, and structure identification of biomolecules remain major objectives in the field of nanopore sensing. Likewise, the shape and size of NPs can be identified with these typical detection principles (Acharya et al., 2020; Si et al., 2020). For example, Karmi et al. used Si_3_N_4_ nanopores to detect gold NPs (AuNP) and differentiate the charged monomers (single particles) and dimers (two particles) based on their different translocation time through the pore (Figure 2J; Karmi et al., 2021). Moreover, the synthesis of NP can be controlled by applying electric fields within a nanopore channel. To achieve this goal, Venta et al. controlled the AuNP synthesis in the SiN_x_ nanopore (sub-10 nm diameter) by driving reactive ions into the nanopore under an applied electric field (Venta et al., 2013). The formation of AuNP was indicated by the characteristic drop in the ionic currents (Figure 2K).

Nucleic acid NP (NANP) is an emerging type of nanostructures with tailorable functions and shapes. Alibakhshi et al. showed that solid-state nanopores could be utilized to characterize and detect (label-free) programmable-shaped NANPs with high precision and efficiency (Alibakhshi et al., 2017). Chen et al. demonstrated that NPs can be applied as efficient readout systems for artificial storage of digital data in DNA nanocarriers (Chen et al., 2019). Moreover, the biomolecular complexes containing DNA-bound proteins, RNA and proteins, 3D structures or DNA, and short, sequence-specific capture probes can also be detected using nanopore-sensing technology. These extra structures will provide characteristic local spikes on top of the ionic current blockade signal (Yang et al., 2018). In addition, the integration of machine learning with solid-state nanopore technology made great progress in the digital detection of viruses at a single-particle level (Arima et al., 2021).

In recent years, solid-state nanopores are gaining popularity for label-free and rapid analysis/detection of NPs due to their diverse applications ranging from medicine to engineering. Nanopores with a combination of resistive pulse technique and advanced fabrication methods can customize pore size to analyze specific NP at an individual level. There is a growing interest in using DNA NPs (nanostructures) for digital data extraction and storage.

## Conclusion

The excellent control over thickness, pore morphology/size, easy functionalization, and stable pore structures, as well as inherent cost and sensitivity/rapidity merits of TEM-fabricated solid-state nanopores have witnessed an exciting future for their applications in single molecule and NP (single-entity) detection. Although these nanopore-based technologies have achieved notable breakthroughs in the past decade, several issues remain to be solved. Issues related to the fabrication of solid-state nanopores need to be fundamentally resolved. TEM-based drilling methods hold diverse opportunities for the fabrication of these nanopores with adjustable sizes, shapes, structures, and atomic resolutions. Keys to success in single-entity detection (small molecules) and efficient scalable technologies are the solid state nanopores better uniformity (size, geometry), manufacturing cost, and stability/reversibility. In this mini-review, we have discussed the latest optimism and progresses on TEM-based fabrication, characterization, and modification of nanopores that are promising in advancing these directions.

Solid-state nanopores can be integrated with tunneling current detection, plasmonic nanopores, and electro–optical-sensing platforms for multiplexed, high-throughput, and multimodalities identification of molecules from a single assay. In addition to device resolution and reliability, some other issues including commercialization, productization, optimization, multiplexing, and mass production of the solid-state nanopore need to be resolved. In-depth understanding of signal-generation mechanisms, designing strategies to enhance the SNR, improving stability and repeatability, engineering membrane structures, and controlling translocation speeds are crucial in designing near-ideal nanopore-based technologies. Accordingly, the incorporation of new nanopore functional and biomolecule modification technologies, the use of specific and sensitive response capturing tools, the introduction of new modalities, and the computer simulation-based analysis could further expand the applications of nanopore sensing. In this regard, further research should be directed toward developing new characterization/detection methods, nanopore materials/membranes, and single-molecule level-sensing applications. Also, combining TEM with multimodal approaches for a comprehensive fabrication/characterization of the nanopores could offer a wonderful opportunity to control and identify the suitable size and geometry (shapes), which urges further exploration.

## Author Contributions

RH conceived and designed the study. All authors conducted a literature survey, drafted and revised the manuscript, and approved it for publication.

## Conflict of Interest

The authors declare that the research was conducted in the absence of any commercial or financial relationships that could be construed as a potential conflict of interest.
